# Exploration of the mechanism of Xinjiang *Rosa laxa* Retz.Fruit extract on IgA nephropathy by bioinformatics and machine learning methods

**DOI:** 10.1186/s40643-026-01090-9

**Published:** 2026-06-17

**Authors:** Feng Qianqian, He Yuan, Guo Ying, Li Aoqing, Dilinur Kamili, Tian Li

**Affiliations:** 1 College of Traditional Chinese Medicine, Xinjiang Medical University, Urumqi, 830017 Xinjiang China; 2 Medical College, Xinjiang University of Science and Technology, Korla, 841000 Xinjiang China; 3 Institute of Traditional Chinese Medicine, Xinjiang Medical University, Urumqi, 830017 China

## Abstract

This study aimed to elucidate the core therapeutic targets and mechanisms of action of rosehip extract (FRLE) in IgA nephropathy (IgAN) by integrating bioinformatics, machine learning, and computational simulation techniques to simultaneously analyze the therapeutic effects of FRLE on IgAN. Through screening differentially expressed genes in IgAN patients and identifying potential target proteins of the core components of FRLE, 118 overlapping targets were ultimately determined as the gene set involved in the synergistic interaction between the two. Through triple machine learning validation, FGR and LDHB were identified as core target proteins, with Ellagic acid, Rutin, and Hyperoside as the core active components. In IgAN patients, LDHB was significantly downregulated, while FGR was significantly upregulated. Both demonstrated excellent diagnostic efficacy and were closely associated with the renal immune microenvironment. Molecular docking and molecular dynamics simulations confirmed that the core components could stably bind to FGR and LDHB. In summary, FRLE targets and modulates FGR and LDHB through its core components, intervening in inflammatory pathways and immune microenvironment imbalance, thereby providing a theoretical basis for subsequent experiments and clinical translation.

**Graphical abstract:**

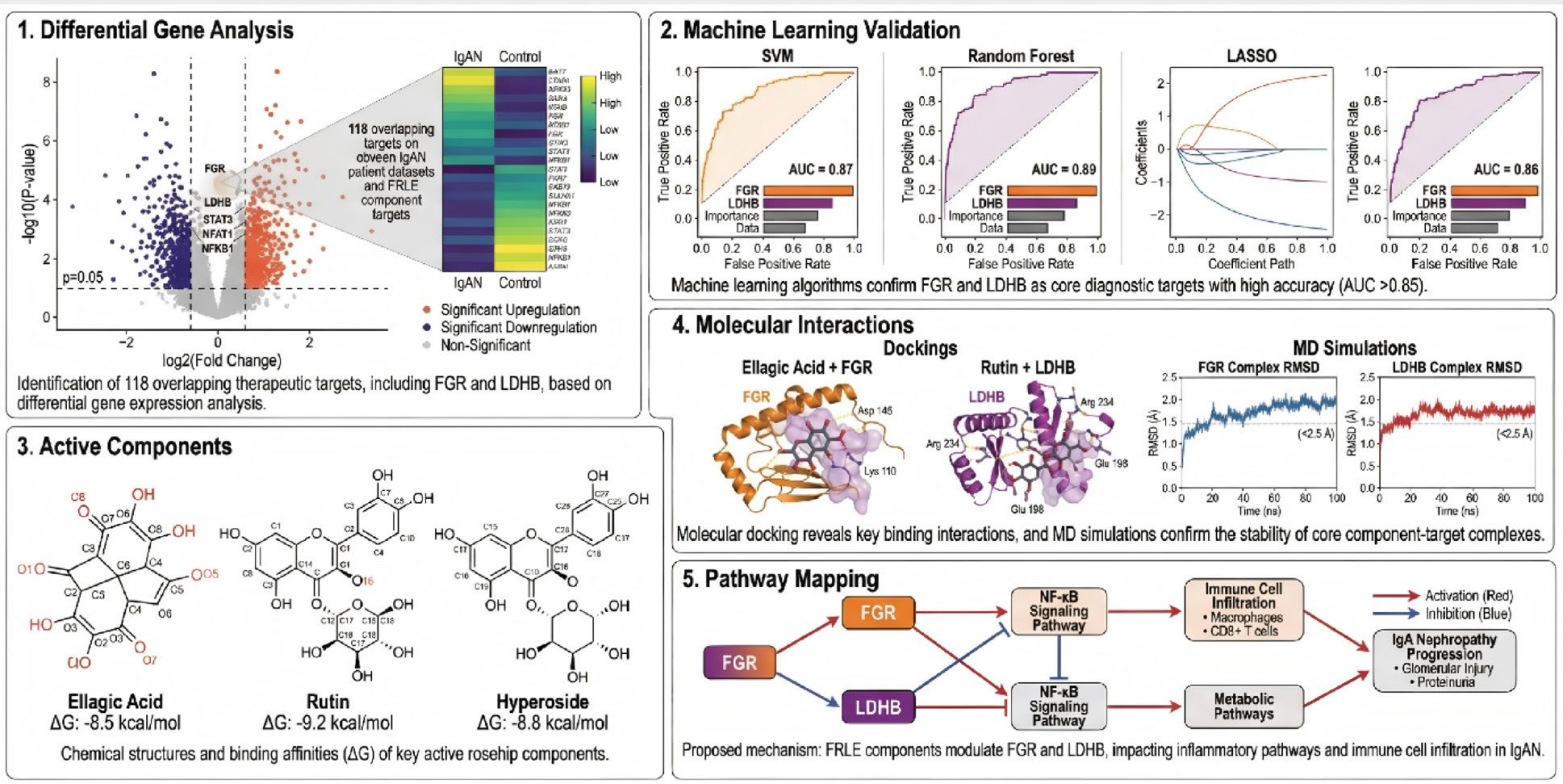

## Introduction

Glomerulonephritis(GN) is one of the leading causes of chronic kidney disease and end-stage renal disease worldwide. Current therapeutic approaches are limited by the severe adverse effects associated with long-term use of corticosteroids and immunosuppressants, as well as suboptimal efficacy in some patients, which drives the search for more suitable alternative treatments (Shankar et al. 2025). Xinjiang Rosa laxa Retz., a plant rich in natural bioactive compounds with a favorable safety profile, is explicitly documented in the Kazakh Materia Medica for its fruit’s therapeutic effect on chronic nephritis. However, the mechanisms by which Rosa laxa Retz. fruit ameliorates GN remain to be elucidated. In this study, bioinformatics and machine learning algorithms were employed to predict the potential core targets of Rosa laxa fruit extract against GN, namely FGR and LDHB. Molecular docking and molecular dynamics simulations were further conducted to validate these key targets, with a focus on evaluating the binding affinity and stability of its key compounds—Ellagic acid, Hyperoside, and Rutin—with the targets. GO and KEGG enrichment analysis results indicate that the therapeutic effects of Xinjiang rose fruit extract may be associated with regulating pathways related to inflammatory responses, immune cell activation, and glomerular mesangial cell proliferation. Molecular docking results show that the binding energy range of two key targets, FGR and LDHB, with key compounds, Ellagic acid, Hyperoside, and Rutin, is −6 to −11 kcal/mol, indicating moderate binding affinity between the target proteins and compounds. This suggests certain binding potential between the target proteins and compounds, which can serve as candidate compounds for further optimization in treating IgA nephropathy (IgAN). Stable binding conformations were also observed from molecular dynamics simulations. Therefore, Ellagic acid, Hyperoside, and Rutin in Xinjiang rose fruit extract may improve pathological damage of glomerulonephritis by inhibiting the activity of FGR and LDHB targets, thereby reducing renal inflammatory infiltration and abnormal mesangial cell proliferation.

Globally, IgA nephropathy (IgAN) is the most common subtype of primary glomerulonephritis, accounting for approximately 30–50% of all primary glomerulonephritis patients. Notably, about 20–30% of IgAN patients progress to end-stage renal disease (ESRD) within 10–20 years after diagnosis, making IgAN one of the core glomerulonephritis subtypes that develop into ESRD (Wheeler et al. 2021).Early diagnosis of IgA nephropathy often involves the frequent oversight of hematuria or proteinuria symptoms. In countries with limited medical resources and inadequate systematic screening mechanisms, the diagnosis of IgA nephropathy is often delayed or misdiagnosed, leading to accelerated disease progression and increased risks of disability and death (Tonelli et al. 2020).The affected population is predominantly young adults aged 20–40 years, with a higher prevalence in males than females. The male-to-female ratio of cases is approximately 2:1, and male patients exhibit a higher risk of disease progression (Ghaddar et al. 2024).The pathogenesis of IgA nephropathy has common mechanisms with the majority of glomerulonephritis; both take immune-mediated glomerular inflammation as their foundation, which is caused by immune complex deposition. However, this disease has its pathological characteristic as abnormal immunoglobulin A (IgA) deposition in the glomerular mesangium. Hence, this deposition will later give rise to mesangial cell multiplication, mesangial stromal enlargement and glomerular structural damage, thus finally causing continuous renal function reduction. At present, glucocorticoids combined with immunosuppressants still serve as the main clinical treatment measure for IgA nephropathy. However, long-time application of such medicines can easily arouse bad effects such as osteoporosis, elevated infection risk, and hepatorenal harm. According to statistical data, about 30% of patients show weak reaction to current standard treatment methods, and certain situations can even not effectively stop disease development, thus displaying obvious limitations in treatment effect.(Zhao et al. 2023).Furthermore, the morbidity mechanism of IgA nephropathy is complicated, which relates to a pathological network connected with immune regulation disorder and metabolic abnormality. Therefore, relying only on single-target medicines often cannot realize effective therapeutic interference, hence this situation shows a limitation of present treatment strategies.Therefore, there is an urgent need for the development of novel therapeutic agents in clinical practice that can precisely target the intricate pathological mechanisms of IgA nephropathy while ensuring both efficacy and high safety. This is of significant importance for the precise modulation of key pathological processes, slowing disease progression, and improving long-term patient outcomes.

FRLE has been documented in traditional medicine for the direct treatment of nephritis, with a common dosage of 50–100 g. Previous research by our group has shown that FRLE is rich in components such as Ellagic acid, Hyperoside, Vitamin C, as well as flavonoids, polyphenols, and triterpenoid acids (Wang et al. 2025). IgAN is characterized primarily by renal immune inflammation triggered by IgA immune complex deposition, abnormal proliferation of mesangial cells, oxidative stress, and renal interstitial fibrosis. Ellagic acid, Hyperoside, and Vitamin C can synergistically regulate this pathological process: Ellagic acid exerts anti-inflammatory effects by inhibiting the STAT3 and NF-κB signaling pathways and exerts anti-fibrotic effects by downregulating the TGF-β1 signaling pathway (Huang et al. 2024). Hyperoside synergistically contributes to anti-inflammatory and immunomodulatory activities, reducing IgA immune complex deposition; vitamin C provides antioxidant support and assists in immune regulation. Together, these three components form a synergistic “anti-inflammatory–anti-fibrotic–antioxidant” system (Dennis et al. 2017). The preliminary research results of the study group indicate that this drug exhibits no significant hepatotoxicity or nephrotoxicity. Therefore, such drugs are more suitable for long-term maintenance therapy in patients with nephritis requiring prolonged medication. This suggests that Rosalaxa fruit has the potential as a natural therapeutic agent for adjuvant treatment of glomerulonephritis, providing a new research direction for optimizing patient prognosis in clinical practice.

A multidimensional research approach was used to study how FRLE regulates the disease process in IgAN. In IgAN, the core problematic phenomenon is the generation of abnormal IgA1 immune complexes. Therefore, these complexes accumulate within the mesangial region of the kidney, thus triggering inflammatory reactions and organ damage. FGR is a crucial enzyme existing in immune cells; hence, it exerts a function throughout the whole disease progression process, starting from the initiation of immune system abnormalities and ending with the final occurrence of kidney impairment. First, FGR controls signals from Fc receptors and integrins. This control affects how immune cells recognize and take in the immune complexes, and it also makes the inflammatory response bigger. Then, it makes mesangial inflammation worse, causes mesangial cells to grow too much, and damages tubules. It does this by turning on pathways like STAT3 and NOTCH1 (Jing et al. 2021). In IgAN patients, the deposition of immune complexes causes a lack of oxygen and inflammation in the kidney. This condition makes LDHB unable to perform its normal function in lactate metabolism. As a result, too much lactate builds up in the kidney. This buildup of lactate then makes the inflammation worse. It also increases damage to the glomerular mesangium. At the same time, it harms the kidney tubules and leads to the growth of fibrous tissue in the kidney. The lactate buildup also affects the metabolism of immune cells. Because of this change, these immune cells start to produce more abnormal IgA1 (Fang et al. 2024).Three core chemical components of FRLE are Ellagic acid, Hyperoside, and Rutin. These compounds all have the ability to target and bind to two key proteins, FGR and LDHB. Molecular docking experiments provided specific binding results. Ellagic acid presented the most powerful binding strength to FGR. Hyperoside exhibited the most stable binding action with LDHB. Rutin showed fine binding capability toward both two proteins. Further kinetic simulations have confirmed the working mechanism of these components. Therefore, they stabilize the structural form of the FGR and LDHB complex; thus, they also alter the functional activity of these proteins. Hence, this enables the components to produce targeted regulatory effects. The combined function of hyperoside and rutin on both FGR and LDHB is of great importance. This joint targeting has direct connection with the inhibition of pro-inflammatory signaling pathways activation. It also has link with the correction of metabolism and immunity imbalance.

This research makes use of bioinformatics technologies for data acquisition, and these technologies supply high-dimensional data coming from genomics, proteomics, and metabolomics levels. Therefore, researchers apply machine learning algorithms to handle this complicated data; the algorithms include Support Vector Machine Recursive Feature Elimination, Least Absolute Shrinkage and Selection Operator regression, and Random Forest. Hence, these methods can carry out accurate identification of key regulatory targets from the datasets, and finding such targets is of great importance for the comprehension of Rosa laxa Retz. fruit extract’s functional mechanisms. One particular algorithm is Support Vector Machine Recursive Feature Elimination: it operates via an iterative process, in which in every step, features with the smallest contribution to disease classification are removed, and this process goes on until it locates the optimal target combination. The Root Mean Square Error is used to measure the prediction accuracy of this model (Sanches et al. 2024).This research utilizes bioinformatics technologies to carry out data acquisition, and these technologies provide high-dimensional data that comes from genomics, proteomics, and metabolomics levels. Therefore, researchers employ machine learning algorithms to deal with this complex data; such algorithms include Support Vector Machine Recursive Feature Elimination, Least Absolute Shrinkage and Selection Operator regression, and Random Forest. Hence, these methods can conduct accurate identification of key regulatory targets from the datasets, and the discovery of such targets has great significance for the understanding of Rosa laxa Retz. fruit extract’s functional mechanisms. Support Vector Machine Recursive Feature Elimination is one specific algorithm: it runs through an iterative process, in which in each step, features that have the smallest contribution to disease classification are removed, and this process continues until it finds the optimal target combination. The prediction accuracy of this model is measured by the Root Mean Square Error (Zhou et al. 2025).

The fast growth of computational chemistry and bioinformatics has pushed computational simulation technologies into natural product drug discovery. Two important examples are molecular docking and molecular dynamics simulation. Molecular docking technology works by predicting the best binding shape of a ligand inside a receptor’s active site. It uses energy calculation parameters to measure how strongly they bind. This provides the main data needed for screening potential therapeutic targets. Molecular dynamics simulation works in a different way. It follows the interaction between receptor and ligand step by step over time. It does this at the atomic level. This method clearly shows how biomolecules change their shape during the process. It also shows how networks of interactions between molecules are formed. And it reveals the main pathways through which they act (Adediwura et al. 2024).Computational simulation techniques have certain advantages over traditional experimental methods. They can perform microscopic analysis at the atomic level. They are also useful in more situations. They are safer to use and cost less money. Traditional experiments have several drawbacks. They need very long time cycles to finish their work. They have extremely high cost levels. Also, they are very difficult to catch dynamic processes at the moment they take place. Therefore, computational techniques can solve these problems in an effective way. Hence, they provide a different method to realize the molecular mechanisms about how drugs do interaction with their target objects.

This research adopts a number of methods to investigate how FRLE could possibly conduct treatment on glomerulonephritis and its working mechanisms; the methods cover bioinformatics, machine learning, and computational simulation. First, bioinformatics analysis searches out potential targets for FRLE, thus helping to confirm which biological targets and signaling pathways may have involvement in the process. Next, through screening huge volumes of biological data, machine learning algorithms select out those core regulatory targets that hold the closest linkage with the disease’s development, and this step is of key importance for the comprehension of FRLE’s working ways. After that, molecular docking technology carries out inspection on the binding degree between FRLE’s main chemical components and these core targets, so as to assess their binding affinity. Finally, molecular dynamics simulations perform verification on the stability of these binding conformations; they construct molecular models and observe the interaction processes between compounds and targets across time, and therefore confirm if the binding possesses stability and specificity.

## Methods

### Data processing and differential expression gene analysis

The data used in our study came from the GEO dataset GSE127136, which is a single-cell RNA sequencing (scRNA-seq) expression profile dataset published in July 2021. It contains renal biopsy samples from 13 IgA nephropathy (IgAN) patients and normal adjacent renal tissues of 6 renal cell carcinoma patients. During sample preparation, glomerular and non-glomerular tissues were distinguished and epithelial cells and macrophages were obtained by using CD326 + and CD14 + MACS technology, while the cells not labeled by MACS were collected to include all kinds of renal cells.

The original raw gene count data coming from GSE127136 dataset were input into R software to conduct analysis; After we utilized the DESeq2 software package to filter genes with low expression, analysis on differential gene expression was then carried out. Genes with significant inter-group expression differences were identified based on corrected *p*-values (padj) < 0.05 and |log2(Fold Change)| ≥1. Results were validated using visualization tools to generate volcano plots of differentially expressed genes. A standardized expression matrix was constructed through variance stabilization transformation (VST). To ensure the reliability of the results, differential expression analysis was repeated using different methods, with the limma software package employed for linear models and empirical Bayesian testing. Gene expression heatmaps were created using the pheatmap software package, visualizing the top five genes with increased expression and the top five genes with decreased expression.

## Potential targets of FRLE against nephritis

Using the PubChem database, we searched for the 12 major compounds identified in FRLE and obtained their simplified SMILES representations. Subsequently, the SMILES representations of these 12 major compounds were uploaded to the SwissTargetPrediction platform (http://swisstargetprediction.ch/). By analyzing the chemical structures of the compounds and integrating existing target information, we efficiently identified potential targets. Through the construction of a Venn diagram, we visualized the overlapping genes between FRLE-related targets and differentially expressed genes (DEGs) in IgAN. This approach revealed targets that are simultaneously involved in the pathological process of IgAN and the pharmacological effects of FRLE. These overlapping targets may represent potential therapeutic targets for FRLE’s anti-nephritis effects.

## GO and KEGG analysis

GO and KEGG analyses of the intersecting genes were performed using the R software package (Yu et al. 2012). This tool is designed for functional enrichment analysis and gene set interpretation, capable of performing functional feature analysis on encoded and non-encoded genomic data from thousands of species, with annotation information sourced from the GO Knowledge Base (Ashburner et al. 2000) and the KEGG pathway database (Kanehisa et al. 2000). Enrichment significance was assessed via a hypergeometric test (using the Benjamini-Hochberg correction method, adjusted *p*-value < 0.05). The hypergeometric test was employed to estimate the probability of observing genes annotated with a specific GO term within the test set. The Benjamini-Hochberg method was applied to control the false discovery rate and correct for multiple testing. The top 10 enriched cellular components, molecular functions, biological processes, and KEGG pathways were visualized using SRplot (Tang et al. 2023).

## GSVA analyse

This study first analyzed the gene expression profiles of the GSE127136 dataset, categorizing the samples into an IgAN group and a NC group to identify DEGs between the two groups. Subsequently, the potential target genes of RLFE compounds were obtained. These targets were intersected with the aforementioned DEGs to derive a core gene set for investigation. Based on this core gene set, GSVA was employed in conjunction with the Hallmark gene sets from the MSigDB database (Liberzon et al. 2015) to calculate pathway activity scores for each sample (Hänzelmann et al. 2013).The differential pathways were subsequently analyzed using the limma package. Pathway changes were assessed by t-values (cut-off value = 1.96, corresponding to *P* < 0.05). Finally, pathway activity heatmaps and differential pathway bar plots were visualized using the pheatmap and ggplot2 packages, respectively.

## Machine learning analysis

Based on the intersection genes, core regulatory genes were screened using three machine learning algorithms, All model constructions and feature selections were performed with fixed random seeds to ensure experimental reproducibility.Lasso regression model was constructed with the glmnet software package (Friedman et al. 2010), The optimal regularization parameter (λ) was selected through 10-fold cross-validation to identify key genes with non-zero coefficients. A random forest classification model was constructed using the randomForest package (Qing et al. 2025). A stable number of decision trees (ntree = 200) was adopted following widely recognized empirical hyperparameters in bioinformatics research, and gene importance was evaluated by the Mean Decrease Gini coefficient. The top 20 core genes were screened and visualized by variable importance bubble plots. The SVM-RFE function was employed to analyze model accuracy and error rates across different feature quantities via 10-fold cross-validation, thereby determining the optimal feature set. Feature ranking bar charts and performance trend plots were generated. Finally, the screening results from the three methods were integrated using a Venn diagram and consistency analysis to validate the reliability of core biomarkers. custom SVM-RFE function was employed, combined with 10-fold cross-validation to analyze model accuracy and error rates across different feature quantities, thereby determining the optimal feature set. Feature ranking bar charts and performance trend plots were also generated. Finally, the screening results from the three methods were integrated using a Venn diagram and consistency analysis to validate the reliability of core biomarkers.

### Differential analysis of core genes, immune infiltration

Based on the identification of two key genes, FGR and LDHB, through three machine learning methods, their expression levels were extracted from the two sample groups. We utilized box plots and violin plots to visualize the expression levels and distribution characteristics of differential genes. The Wilcoxon rank sum test was employed to analyze whether the intergroup differences were statistically significant. Additionally, the receiver operating characteristic (ROC) curve was plotted, and the area under the curve (AUC) along with its 95% confidence interval were calculated to evaluate diagnostic efficacy. Single-sample gene set enrichment analysis (ssGSEA) was employed to compute immune cell infiltration scores. Spearman correlation analysis was used to investigate the association between FGR and LDHB expression and immune cell infiltration, with results visualized via heatmaps and scatter plots (Ren et al. 2025). Finally, immunohistochemical images from the Human Protein Atlas (HPA) database were utilized to validate differences in protein expression of the two genes between normal and diseased renal tissues.

## Molecular docking

The three main 3D structures of Ellagic acid, Hyperoside, and Rutin in FRLE were retrieved and downloaded using the PubChem database. The ligand-protein files were processed with AutoDockTools 1.5.7 software, including ligand hydrogenation and docking type selection, and the final ligand files were exported in PDBQT format (Che et al. 2024). Utilize D3Pocket (https://www.d3pharma.com) to predict and identify potential binding pockets of receptor proteins, score these pockets, and select regions with higher pocket scores as targets for subsequent molecular docking. Perform molecular docking using Autodock Vina, adopting the conformation with the lowest binding energy from each receptor-ligand docking result as the stable conformation to comprehensively evaluate ligand conformation and its interaction with the protein binding pocket. Finally, visualize the docking results using PyMOL software to intuitively demonstrate the interaction patterns between the ligand and the receptor protein.

## Molecular dynamics simulation

Based on the optimal binding conformations of Ellagic acid, Hyperoside, and Rutin with the target proteins FGR and LDHB obtained through molecular docking, we performed molecular dynamics simulations of the optimal conformations using the Gromacs software. The specific steps included ligand/protein preprocessing, complex construction, system solvation, ion neutralization, energy minimization, and NVT/NPT equilibrium, followed by a 20-nanosecond dynamics simulation. Multidimensional trajectory analyses, including RMSD, RMSF, and FES, were employed to validate the dynamic binding stability and interaction patterns of the complexes (Yuan et al. 2025).

## Result

### Prediction of biological targets of FRLE in the treatment of lgAN

**Fig. 1:**
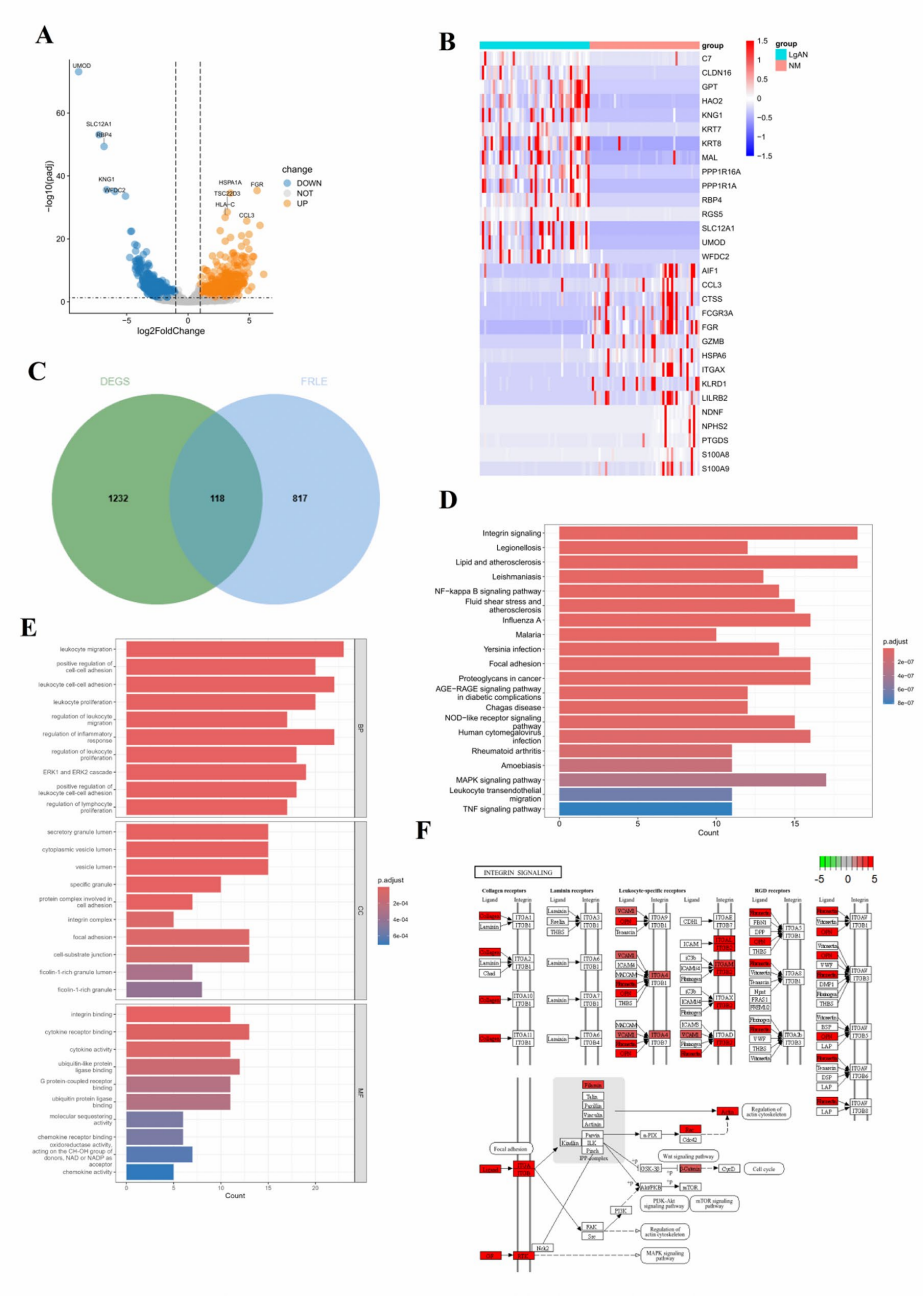
Analysis of differential genes, target protein intersections, and functional enrichment following FRLE intervention in IgA nephropathy. **A**: Volcano plot of differentially expressed genes (DEGs) in IgA nephropathy; **B**: Heatmap showing the expression of core differentially expressed genes in the normal group (NM) versus the IgAN group; **C**: Venn diagram illustrating the intersection between IgA nephropathy DEGs and potential target proteins of RLFE; **D**: Bar chart of KEGG pathway enrichment analysis for the overlapping target proteins; **E**: Bar chart of GO functional enrichment analysis for the overlapping target proteins; **F**: Visualization of the integrin signaling pathway)

The volcano plot visually presents the magnitude of gene expression changes and their statistical significance, providing a systematic summary of the distribution characteristics of DEGs. A total of 1350 DEGs were identified, comprising 721 upregulated genes and 629 downregulated genes (Fig. 1A). A heatmap was used to display the top 15 upregulated and downregulated genes (Fig. 1B). The SwissTargetPrediction platform was employed to predict the target proteins of the core chemical components in FRLE, including Tilianin, Hyperoside, Quinic acid, Rutin, oleanolic acid, Ellagic acid, Kaempferol, Kaempferol-3-O-rutinoside, Euscaphic acid, Isoquercitrin, Pomolic acid, and Astragalin. This analysis identified 817 potential target proteins. Cross-referencing these target proteins with the DEGs associated with IgA nephropathy revealed 118 overlapping targets (Fig. 1C).

Functional enrichment analysis of the 118 overlapping target proteins indicated their primary enrichment in inflammation- and immunity-related pathways, such as the integrin signaling pathway and the NF-κB signaling pathway (Fig. 1D). The integrin signaling pathway contained the highest number of enriched target proteins, establishing it as one of the core pathways through which FRLE intervenes in IgA nephropathy. Through analysis of the GO result(Fig. 1E), it was demonstrated that on the biological process level, these overlapping genes participated in immune-inflammatory processes, for instance leukocyte migration and inflammatory response regulation. At the level of cellular component, these overlapping genes were mainly positioned at adhesion-related constructions such as integrin complexes. Therefore, at the molecular function level, these overlapping genes mostly carried out regulation of signal transduction via integrin binding and cytokine receptor binding. (Fig. 1F).

**Fig. 2:**
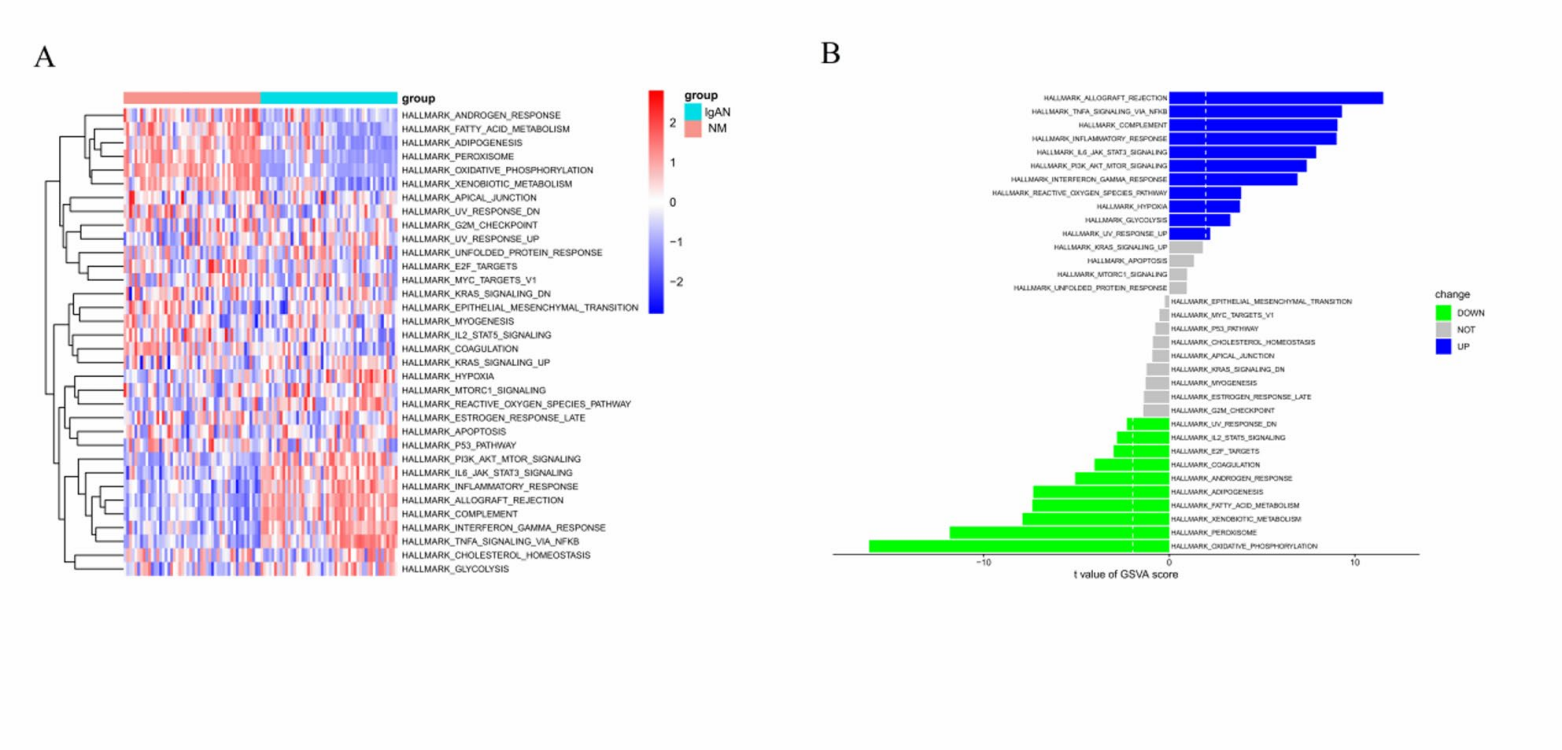
Differences in GSVA pathway activity between the IgAN group and the normal group (NM) (**A**: Heatmap of pathway activity in the IgAN group versus the normal group (NM); **B**: Plot of GSVA pathway enrichment results for IgAN samples)

Validation results from Gene Set Variation Analysis (GSVA) of patient transcriptomic data showed significant upregulation of inflammatory pathways, including the NF-κB pathway, in IgA nephropathy samples, which strongly corroborated the target protein enrichment findings. The final results indicate that the active components of FRLE can exert a potential therapeutic effect on IgA nephropathy by targeting and modulating the integrin signaling pathway and its downstream inflammatory pathways, such as NF-κB and MAPK, thereby correcting the pathway imbalances in patients (Fig. 2A and B).

### FGR and LDHB are core target proteins

**Fig. 3:**
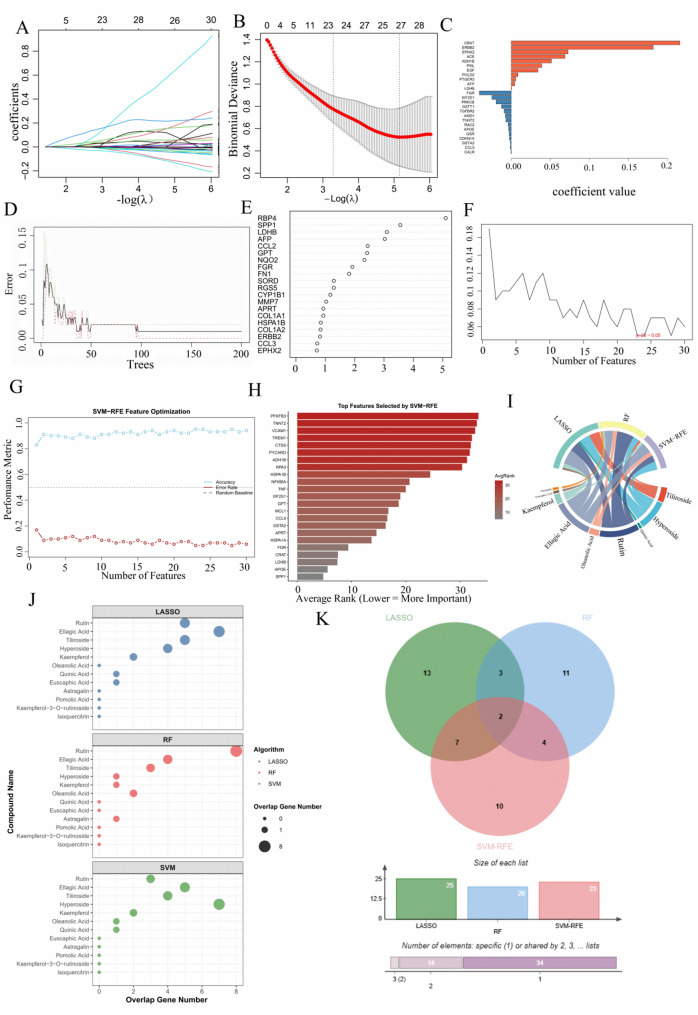
Screening of core targets for IgA nephropathy and visualization of component-target associations based on LASSO regression, random forest, and SVM-RFE. (**A**: LASSO regression coefficient trajectory plot; **B**: LASSO cross-validation deviance curve; **C**: Coefficient distribution of features selected by LASSO; **D**: Random Forest error curve as a function of the number of features; **E**: Random Forest feature importance ranking plot; **F**: Model performance curve during the SVM-RFE feature elimination process; **G**: SVM-RFE cross-validation performance curve; **H**: Top feature ranking plot selected by SVM-RFE; **I**: Chord diagram illustrating the association between core active components and the screened targets; **J**: Heatmap showing the associations between targets screened by different algorithms and active components; **K**: Venn diagram of targets screened by the three algorithms)

To precisely identify the core active components and key targets of FRLE in intervening in IgAN, three machine learning algorithms—LASSO regression, RF, and SVM-RFE—were employed for screening and validation. LASSO regression determined the optimal parameters via coefficient trajectory plots and deviance curves, screening out 25 core features (Fig. 3A, B and C). SVM-RFE and Random Forest were further applied for validation: the former screened 20 optimal features (Fig. 3D and E), while the latter selected 23 optimal feature targets (Fig. 3F, G, and H), all of which pointed to inflammation- and adhesion-related proteins. A Venn diagram of the multi-algorithm cross-validation revealed that two core targets—FGR and LDHB—were identified as the common intersection among the three algorithms (Fig. 3K). By intersecting the active components of FRLE with the key genes obtained from the three algorithms, the core components in FRLE for treating IgAN were analyzed. The results indicated that Ellagic acid, Rutin, and Hyperoside exhibited the closest associations with the core targets screened by each algorithm (Fig. 3I and J). This approach precisely pinpointed the core active components and key targets of FRLE in intervening in IgAN, providing a clear material basis and target direction for subsequent in vitro and in vivo experimental validation.

### Differential analysis of core target proteins

**Fig. 4:**
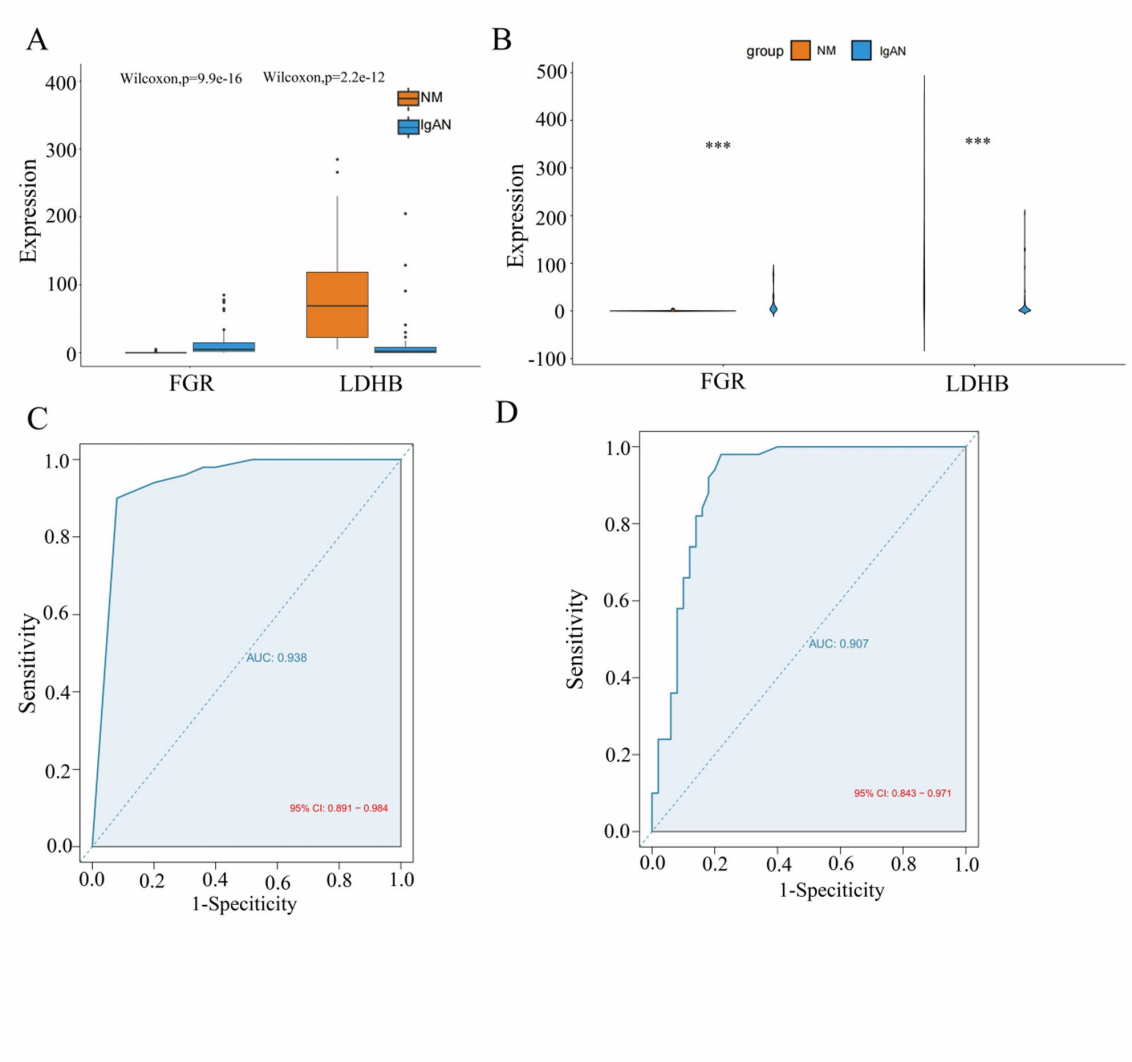
Expression differences and diagnostic performance evaluation of the core target proteins FGR and LDHB in IgA Nephropathy (**A**: Box plot of FGR and LDHB expression in the IgAN group and the NM group; **B**: Violin plot of FGR and LDHB expression in the IgAN group and the NM group; **C**: Receiver operating characteristic (ROC) curve for LDHB; **D**: Receiver operating characteristic (ROC) curve for FGR)

Through differential expression analysis between patients with IgA nephropathy and normal controls, it was found that the core gene LDHB was significantly downregulated in samples from IgA nephropathy patients, while FGR was significantly upregulated (Fig. 4A and B). Further diagnostic efficacy evaluation revealed that both LDHB (AUC = 0.938) and FGR (AUC = 0.907) exhibited excellent disease-discriminating ability (Fig. 4C and D). These findings not only confirm the relevance of these core targets in IgA nephropathy but also suggest their potential as diagnostic biomarkers. Furthermore, they provide supporting evidence for the mechanism by which FRLE exerts its therapeutic effects, namely by targeting and modulating these core inflammatory targets to correct pathway imbalances.

### Regulatory Role of Immunomicroenvironment and Protein Level Verification of Core Target FGR and LDHB

**Fig. 5:**
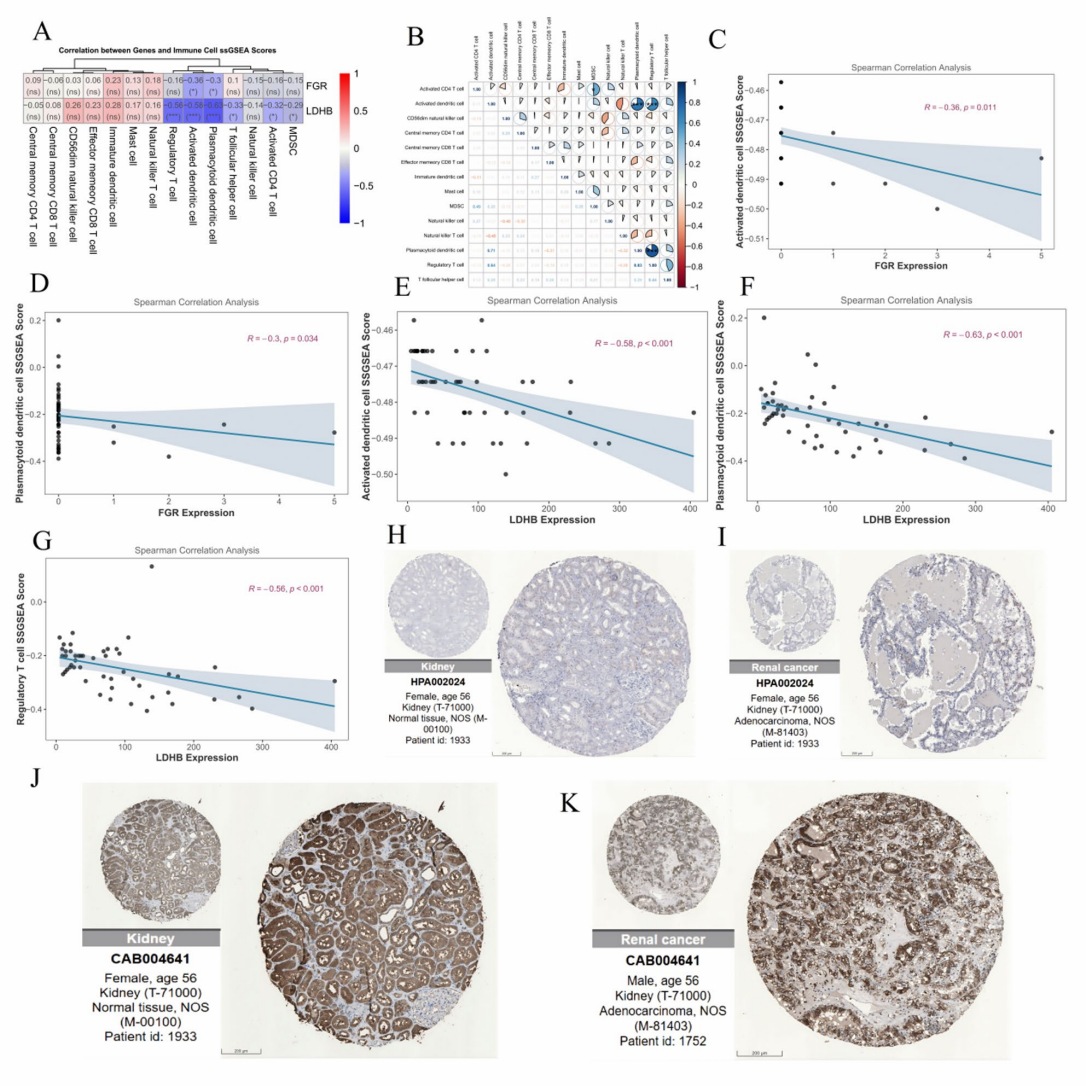
Association of core target proteins FGR and LDHB with the immune microenvironment in IgA nephropathy and validation at the protein level (**A**: Correlation heatmap of core genes (FGR, LDHB) with ssGSEA scores for immune cell infiltration; **B**: Correlation heatmap of immune cell infiltration levels; **C**-**D**: Correlation between FGR expression and infiltration scores of activated dendritic cells and plasmacytoid dendritic cells, quantifying the association strength; **E**–**G**: Correlation between LDHB expression and infiltration scores of activated dendritic cells, plasmacytoid dendritic cells, and regulatory T cells; **H**-**I**: Protein expression levels of FGR in normal kidney tissue versus kidney lesion tissue (adenocarcinoma); **J**-**K**: Protein expression levels of LDHB in normal kidney tissue versus kidney lesion tissue)

Based on the differential expression characteristics of the aforementioned core targets FGR upregulated in IgA nephropathy and LDHB downregulated in IgA nephropathy, their association with the immune microenvironment was further investigated and validated at the protein level: The gene–immune cell correlation heatmap (Fig. 5A) revealed that both FGR and LDHB were significantly negatively correlated with the infiltration levels of immune cells such as activated dendritic cells and plasmacytoid dendritic cells. Combined with the background information from the inter-immune cell correlation heatmap (Fig. 5B), this suggests that both may be involved in the inflammatory process by regulating immune cell infiltration.

Compared with the normal control group, FGR expression was significantly upregulated in the IgAN patient group, while the ssGSEA infiltration scores for activated DCs/plasmacytoid DCs showed a downregulated trend. This suggests that the IgAN samples may predominantly consist of patients in the chronic stage, where prolonged inflammation leads to DC functional exhaustion, and the body initiates excessive compensatory mechanisms to suppress uncontrolled inflammation. Within the IgAN patient group, Spearman correlation analysis demonstrated a significant negative correlation between FGR expression and the infiltration scores of activated DCs/plasmacytoid DCs (*r*=-0.38/-0.30, *p* < 0.05), further validating this compensatory regulatory feature (Fig. 5C and D).

Compared with the normal control group, LDHB expression was significantly downregulated in the IgAN patient group. This result is consistent with the metabolic reprogramming characteristics under the chronic inflammatory state of IgAN and represents an active mechanism by which the body adapts to immune dysregulation through the regulation of lactate metabolism (Luo et al. 2026).Within the IgAN patient group, Spearman correlation analysis showed that LDHB expression was strongly and significantly negatively correlated with the ssGSEA infiltration scores of activated DCs, plasmacytoid DCs, and regulatory T cells (*r* = −0.58, −0.63, −0.56, all *p* < 0.001). The strength of these associations far exceeded that observed for FGR, suggesting that LDHB is a central hub for immune regulation in IgAN (Fig. 5E–G).

Utilizing immunohistochemistry data (HPA: https://www.proteinatlas.org/) from the database (Fig. 5H–I), (antibody IDs: HPA002024, CAB004641), we analyzed two independent sets of LDHB immunohistochemical validation data from the HPA database (HPA002024, CAB004641). The results corresponding to HPA002024 showed weak positive staining for FGR in normal kidney tissue, whereas moderate to strong positive staining was observed in renal carcinoma tissue. In the results corresponding to CAB004641, LDHB exhibited strong positive staining (dark brown) in normal kidney tissue, which was significantly attenuated (light brown/pale staining) in renal carcinoma tissue (Fig. 5J-K). Both independent validation results consistently confirmed a significant upregulation of FGR protein expression in renal lesion tissues. This finding not only corroborates the conclusions from the transcriptomic data at the protein level but also provides reliable multi-dimensional evidence supporting the mechanism by which FRLE targets and regulates LDHB to intervene in renal inflammatory injury.

### Core chemical components and molecular docking results of FGR and LDHB

**Fig. 6:**
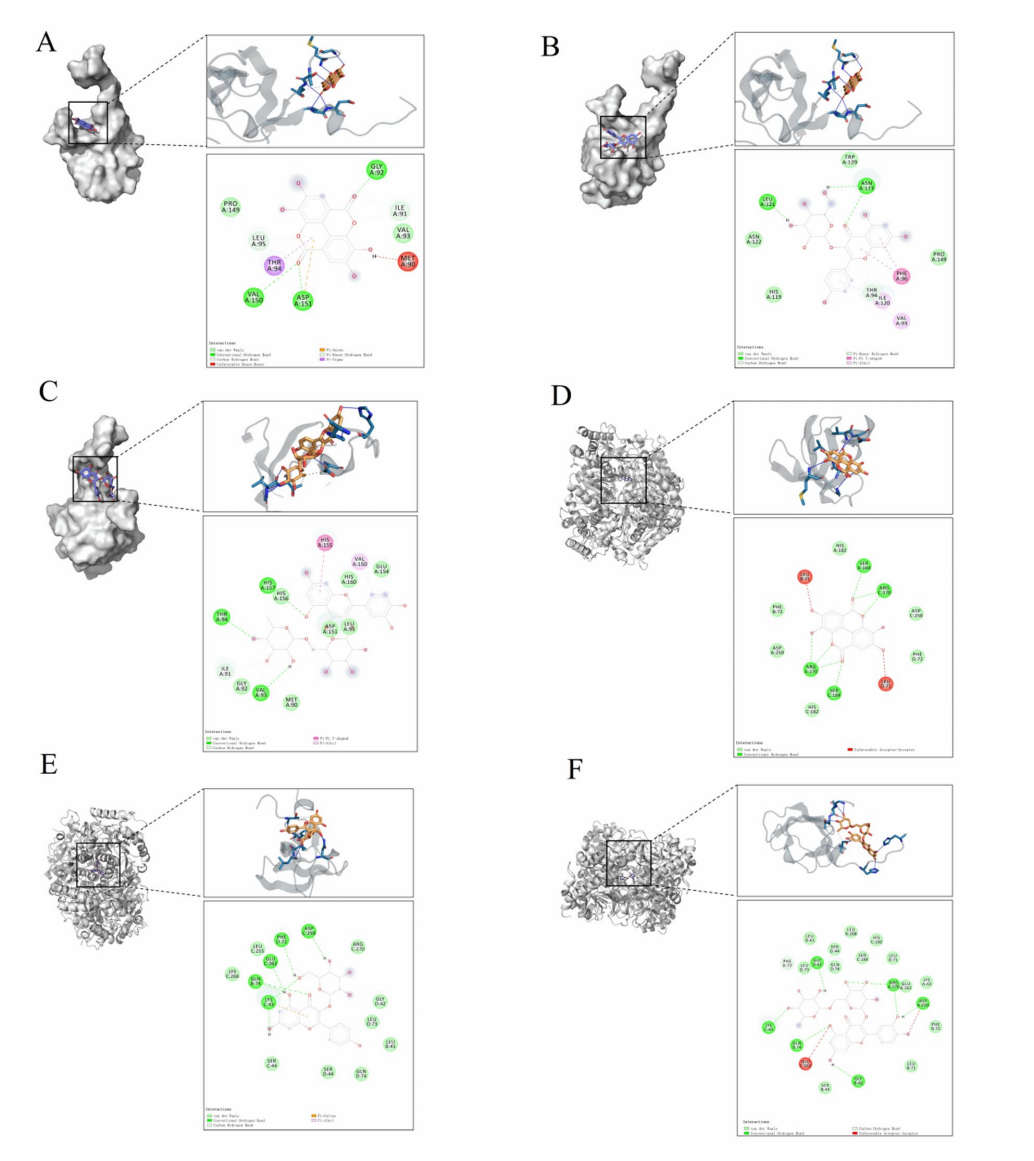
Molecular docking binding modes of FRLE core components with FGR and LDHB (**A**: Binding mode of Ellagic acid with FGR; **B**: Binding mode of Hyperoside with FGR; **C**: Binding mode of Rutin with FGR; **D**: Binding mode of Ellagic acid with LDHB; **E**: Binding mode of Hyperoside with LDHB; **F**: Binding mode of Rutin with LDHB)

Analysis of the molecular docking results for Ellagic acid, Hyperoside, and Rutin with FGR revealed distinct differences in their binding potential to the FGR kinase. Ellagic acid (Fig. 6A). Rutin (Fig. 6C) binds through its sugar part working together with its main flavonoid part. This combined action allows it to bind in a stable way, and it has the second strongest binding potential. Hyperoside (Fig. 6B) binds using only hydrogen bonds and van der Waals forces. It has the least stable binding among them.

Analysis of the molecular docking results for Ellagic acid, Hyperoside, and Rutin with LDHB also indicated significant differences in binding stability (Table 1) Hyperoside (Fig. 6E) made the most stable binding network with LDHB. It uses hydrogen bonds, π-cation interactions, and van der Waals forces. No bad interactions were seen. Rutin (Fig. 6F) has the second most stable binding. Its two-sugar structure gives many polar spots for binding. It uses hydrogen bonds and van der Waals forces. But there is one weak bad acceptor-acceptor interaction. Ellagic acid (Fig. 6D) binds with the least stability. It forms hydrogen bonds and van der Waals interactions. But two bad acceptor-acceptor interactions happen with it.

**Table 1:** Table of molecular docking energy values of compounds and targets

	LDHB	FGR
Hyperoside	−8.7 kcal/mol	−6.1 kcal/mol
Ellagic acid	−9.1 kcal/mol	−6.1 kcal/mol
Rutin	−10.6 kcal/mol	−6.5 kcal/mol

### Molecular dynamics simulation of core chemical components and FGR and LDHB

**Fig. 7:**
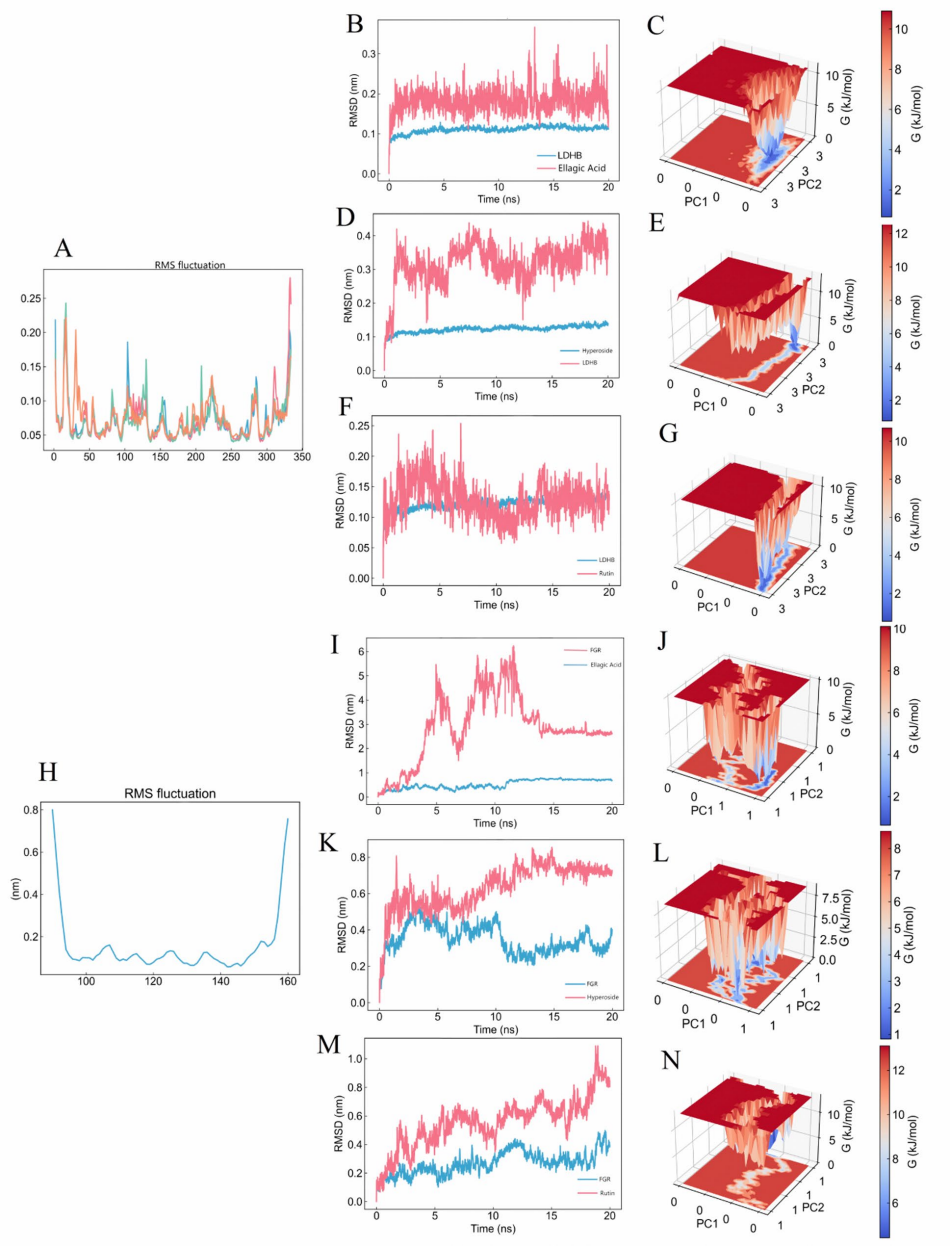
Stability analysis of molecular dynamics simulations for the complexes formed between FRLE core components and FGR or LDHB. (**A**: RMS fluctuation curve of LDHB protein residues; **B**: RMSD curve of the LDHB–Ellagic acid complex; **C**: PCA conformational distribution plot of the LDHB–Ellagic acid complex; **D**: RMSD curve of the LDHB–Hyperoside complex; **E**: PCA conformational distribution plot of the LDHB–Hyperoside complex; **F**: RMSD curve of the LDHB–Rutin complex; **G**: PCA conformational distribution plot of the LDHB–Rutin complex; **H**: RMS fluctuation curve of FGR protein residues; **I**: RMSD curve of the FGR–Ellagic acid complex; **J**: PCA conformational distribution plot of the FGR–Ellagic acid complex; **K**: RMSD curve of the FGR–Hyperoside complex; **L**: PCA conformational distribution plot of the FGR–Hyperoside complex; **M**: RMSD curve of the FGR–rutin complex; **N**: PCA conformational distribution plot of the FGR–rutin complex)

These figures present the molecular dynamics simulation results of proteins LDHB and FGR with Ellagic acid, Hyperoside, and Rutin, respectively, focusing on the conformational stabilizing effects of the ligands on the protein complexes (Fig. 7A–N). After the binding of rutin with LDHB, the RMSD curve exhibited a stable trend, and the PCA conformation showed a highly aggregated state, indicating excellent conformational stability. The binding of hyperoside with LDHB also effectively reduced conformational fluctuations, demonstrating comparable binding stability. For FGR, after binding with hyperoside or rutin, the RMSD fluctuations are significantly reduced, and the clustering of PCA conformations is markedly enhanced; both ligands show outstanding stabilizing effects on the FGR complex. In summary, rutin exhibits the optimal binding stability with LDHB, while hyperoside and rutin show similar binding stability with FGR. Both ligands can efficiently enhance the conformational stability of the LDHB and FGR complexes, demonstrating excellent binding performance.

## Conclusion

The pathological core of IgAN is a complex network interwoven with immune dysregulation and metabolic imbalance. Nowadays, main clinical treatment plans first pay attention to symptom easing and functional support, and they do not directly aim at the disease’s root reason or occurrence mechanism.(Liu et al. 2026). Natural products have intrinsic advantages of “multi-component, multi-target, and synergistic regulation.” These advantages make them an important research field for IgAN therapy. In detail, this study displayed how FRLE acts at molecular level to interfere with IgAN. Therefore, thus, the research findings prove FRLE’s various characteristics; these characteristics indicate its special potential as a natural product for IgAN treatment.

The “multi-component, multi-target” action mode of FRLE matches with the complex pathological mechanisms of gAN. Previous studies (by relevant researchers, year unknown) have identified 12 key active components inside FRLE, which are capable to retrieve 817 potential proteins. Therefore, by making intersection between these 817 potential proteins and the differentially expressed genes in IgAN, we can obtain 118 overlapping proteins. Hence, analysis result shows that these overlapping proteins are enriched in core inflammatory and immune pathways such as integrins, NF-κB, and MAPK. These results are consistent with the pathological process of IgAN, which involves “immune activation, adhesion abnormalities, and inflammatory damage.” Multiple components in FRLE can synergistically regulate the excessive growth of immune cells and mesenchymal cells, as well as the release of inflammatory factors, thereby fundamentally correcting the pathway abnormalities in IgAN. This “multi-component, multi-target” action mode represents a unique advantage of natural products and provides a new direction to address the challenge of “limited efficacy of single-intervention approaches” in the treatment of IgAN.

This research screened some potential key genes by using several machine learning methods and then determined the candidate targets FGR and LDHB through FRLE analysis. In IgA nephropathy, FGR was found to be overexpressed whereas LDHB had lower expression, showing a distinct expression pattern. The ROC analysis indicated that FGR and LDHB have good discriminative power for sample classification with AUC values of 0.907 and 0.938 respectively, suggesting their possible value as diagnostic markers.

In patients with IgAN, the expression of FGR is upregulated abnormally while that of LDHB is significantly downregulated, indicating an opposite expression imbalance. Our study has shown that the main components of FRLE—ellagic acid, hyperoside and rutin—can specifically act on these two opposite changes: on one hand, ellagic acid binds to the overexpressed FGR with high affinity, and together with hyperoside and rutin, they can stabilize the structure of FGR and thus inhibit its overactivated pro-inflammatory signal and reduce the inflammatory damage caused by FGR; on the other hand, hyperoside and rutin can also stabilize the conformation of LDHB, reverse its suppressed state and restore its metabolic and immune homeostatic functions. Therefore, instead of merely promoting or inhibiting gene expression in a single direction, FRLE exerts a special reverse regulatory action according to the diseased expression imbalance: it inhibits the overactivated inflammatory factors and restores the suppressed homeostatic factors, finally and continuously restoring the immune and metabolic homeostasis in the kidney of IgAN patients. This “multi-component bidirectional correction mechanism” also clarifies the possible advantages of FRLE in regulating the complicated inflammatory network of IgAN.

The active components with medicinal value in natural products are mostly extracted from plants that have undergone prolonged artificial selection and practical validation. These plants are typically those that have been used and screened over time in traditional medicine or folk practices, demonstrating certain efficacy and high safety profiles for human use.(Xie et al. 2025). As a natural product, FRLE holds promising prospects in clinical applications and other fields.In comparison with single-drug therapy, natural products hold greater suitability for long-term pharmacological interference in chronic kidney disease patients (CKD). Based on the research results of this study, rosehip fruit shows pharmacological capabilities including anti-inflammation, anti-oxidation, and kidney protection effects. FRLE can aim at key targets FGR and LDHB to interfere in the pathological progress of immune dysregulation and metabolic disorder in IgA nephropathy (IgAN); therefore, thus it realizes a triple function of “anti-inflammation, immune regulation, and metabolic control.”

This research used a mixed investigation method that combines high-throughput sequence screening, machine learning verification, molecular docking, and kinetic simulation to explain FRLE’s treatment mechanism in IgAN. Therefore, the research found FRLE’s core active components and key targets that affect IgAN, meanwhile, it also provides a method framework for mechanism researches of other natural products. Hence, the built standards for FRLE’s targeted regulation of core targets FGR and LDHB supply possible diagnostic chances for IgAN, thus showing clinical application value to realize precise treatment in this disease.

This study also has certain limitations. The direct treatment effects of FRLE on IgAN and the cooperative effects of its core parts have not received validation via in vitro and in vivo tests. Therefore, future research must center on performing related cell experiments to verify FRLE’s influence on FGR and LDHB expression, as well as its adjustment effects on inflammatory pathways. We must here point out that IgAN in its essence is one inflammatory non-malignant disease. The utilization of malignant tumor tissue protein information as a substitute for IgAN protein level verification has some certain limitations. The molecule-related background of tumor tissue has big differences from the long-term inflammation microenvironment of IgAN kidney damage, which hence may bring possible deviation in biomarker expression modes. Therefore, the current protein verification outcomes are only for preliminary reference use, and thus follow-up confirmation based on clinical IgAN kidney samples is necessary. Hence, in addition, animal experiments should be implemented to build an IgAN animal model for assessing its kidney protection effects; after that, preclinical safety evaluations must be done, thus laying a more firm basis for clinical translation.In summary, as a natural product, FRLE demonstrates significant potential value in IgAN treatment by virtue of its unique advantages: “multi-component synergy, multi-target precision, adaptation to pathological complexity, and good biocompatibility.” This study systematically elucidated its mechanism of action, providing a new theoretical basis for the development and utilization of natural products and opening a new direction for the precise treatment of IgAN.

## Data Availability

The datasets used and/or analyzed during the current study are available from the corresponding author on reasonable request.

## Abbreviations

RLFE: Rosa laxa Retz.Fruit extract
IgAN: IgA nephropathy
GN: Glomerulonephritis
ESRD: End-stage renal disease
IgA: Immunoglobulin A
SVM-RFE: Support vector machine recursive feature elimination
LASSO: Least absolute shrinkage and selection operator
RF: Random forest
RMSE: Root mean square error
DEGs: Differentially expressed genes
VST: Variance-stabilizing transformation
ROC: Receiver operating characteristic
AUC: Area under the curve
ssGSEA: Single-sample gene set enrichment analysis
HPA: Human protein atlas
